# Extracellular Vesicles from Hypoxic Adipocytes and Obese Subjects Reduce Insulin‐Stimulated Glucose Uptake

**DOI:** 10.1002/mnfr.201700917

**Published:** 2018-02-20

**Authors:** Justyna Mleczko, Francisco J. Ortega, Juan Manuel Falcon‐Perez, Martin Wabitsch, Jose Manuel Fernandez‐Real, Silvia Mora

**Affiliations:** ^1^ Department of Molecular and Cellular Physiology Institute of Translational Medicine The University of Liverpool Liverpool UK; ^2^ CIC bioGUNE CIBERehd Derio Bizkaia Spain; ^3^ Dept Diabetes Endocrinology and Nutrition Institut Investigacio Biomedica Girona (IdibGI) Girona Spain; ^4^ CIBER Obesidad (CIBERObn) Instituto de Salud Carlos III Madrid Spain; ^5^ IKERBASQUE Basque Foundation for Science Bilbao Bizkaia Spain; ^6^ Division of Pediatric Endocrinology and Diabetes Department of Pediatrics and Adolescent Medicine University of Ulm Germany

**Keywords:** adipocyte, exosome, glucose transport, insulin

## Abstract

**Scope:**

We investigate the effects of extracellular vesicles (EVs) obtained from in vitro adipocyte cell models and from obese subjects on glucose transport and insulin responsiveness.

**Methods and results:**

EVs are isolated from the culture supernatant of adipocytes cultured under normoxia, hypoxia (1% oxygen), or exposed to macrophage conditioned media (15% v/v). EVs are isolated from the plasma of lean individuals and subjects with obesity. Cultured adipocytes are incubated with EVs and activation of insulin signalling cascades and insulin‐stimulated glucose transport are measured. EVs released from hypoxic adipocytes impair insulin‐stimulated 2‐deoxyglucose uptake and reduce insulin mediated phosphorylation of AKT. Insulin‐mediated phosphorylation of extracellular regulated kinases (ERK1/2) is not affected. EVs from individuals with obesity decrease insulin stimulated 2‐deoxyglucose uptake in adipocytes (*p* = 0.0159).

**Conclusion:**

EVs released by stressed adipocytes impair insulin action in neighboring adipocytes.

## 1 Introduction

White adipose tissue (WAT) regulates glucose and lipid homeostasis and functions as an endocrine tissue releasing adipokines that contribute to regulate insulin sensitivity. White adipocytes respond to insulin by activating glucose transport through the translocation and activation of the glucose transporter GLUT4.^1^ Excess glucose is converted to glycerol for esterification of fatty acids into triglycerides, which are later hydrolyzed to provide fatty acids during fasting. In obesity adipose cells fail to respond to insulin and glucose transport and lipid regulation are impaired. During WAT expansion, cells are exposed to local hypoxia which contributes to the dysregulation of production and secretion of adipokines.^2^ Adipocytes are also exposed to pro‐inflammatory cytokines following the recruitment, infiltration, and activation of macrophages within WAT, which in turn further impair insulin action.

In recent years the importance of a novel mechanism for intercellular communication has emerged based on the release of extracellular vesicles (EVs).^3^ EVs of various sizes, namely exosomes (with sizes between 30–150 nm), microvesicles (ranging from 100–1000 nm) or apoptotic bodies (ranging 150–5000 nm) are released by eukaryotic cells. EVs contain protein and RNA species that regulate physiological functions and gene expression in target cells^4^ eliciting both autocrine and paracrine effect in various cell types. EV composition and release are modified in pathological conditions, with EVs serving as biomarkers for human disease.^5^, ^6^

Adipocytes have been shown to produce EVs of heterogeneous sizes^7^, ^8^, ^9^, ^10^ which contribute to regulate lipid deposition^11^, ^12^ and angiogenesis,^13^ reviewed in.^14^ Deng et al.^15^ were the first to report that EVs from adipose tissue explants from obese (*ob/ob*) mice induced insulin resistance in vivo when injected into wild type mice.^15^ Despite the available data obtained in animal and in vitro studies, adipocyte‐derived EVs remain poorly characterized and limited information is available on their effects in normal and pathophysiological conditions.

This study aimed at determining the impact of adipose‐released EVs on insulin action. Specifically, we tested the hypothesis that EVs released by stressed adipocytes and those found circulating in human obesity impact on insulin‐stimulated glucose uptake.

## 2 Experimental Section

### 2.1 Materials Reagents and Antibodies

Tissue culture media and reagents were from Sigma–Aldrich (UK). B‐tubulin antibody was from Sigma–Aldrich, Phospho‐Ser473 AKT, total AKT, phospho44p42, and total ERK (p44/p42) antibodies were from Cell Signalling. Anti‐CD81 antibody was from AD Serotec (UK), anti‐TSG101 (Ab83), anti‐MHCI (clone 1158Y) anti‐beta actin (Ab6276), anti‐catalase (Ab15834), and anti‐GAPDH (Ab9484) antibodies were from Abcam (UK). Anti‐VEGF (VG‐1) antibody was from Santa Cruz Biotechnology.

### 2.2 Cell Culture

Human Simpson–Golabi–Behmel syndrome (SGBS) cells were grown and differentiated as described.^16^ 3T3‐L1 cells were cultured and differentiated as described.^17^ Prior to the isolation of EVs from the conditioned media, cells were washed in PBS and cultured in DMEM without any serum supplementation for 24 h. For the macrophage conditioned media treated group, DMEM was supplemented with 15% v/v of media from Bone Marrow Derived Macrophages cells. Isolation of Bone Marrow Derived Macrophages cells was carried out as described.^18^ This media contained on average 195 ng mL of IL6 and 150 ng mL^–1^ of TNFα quantified by ELISA. For the hypoxia experiments, 3T3‐L1 adipocytes were incubated in normoxia (95% air, 5% CO_2_) or inside an hypoxic chamber (hypoxystation1% O_2_, 5% CO_2_, and 95% N_2_) for 24 h. EV treatments were carried out in DMEM without supplementation.

### 2.3 Subjects

Plasma was obtained from *n* = 8 lean and from *n* = 9 women with obesity recruited to the Endocrinology Service of the Hospital Universitari Dr. Josep Trueta (Girona, Spain), and were used for EVs isolation. Exclusion criteria were: abnormal blood counts and liver, kidney, or thyroid dysfunction, evidence of chronic illness other than obesity, chronic use of medication, acute illness, or signs of infection in the month preceding enrolment. The study protocol was approved by the Ethics Committee and the Committee for Clinical investigation (CEIC) of the Hospital Universitari Dr. Josep Trueta. All subjects gave their written informed consent. Plasma samples kept at –80 °C until the isolation of EVs. Fasting glucose levels were measured by the glucose oxidase method with a Beckman Glucose Analyzer 2 (Brea, CA). Plasma insulin was determined by ELISA using a commercial kit (Catalog E6000‐K, Millipore, USA) following the manufacturer's instructions.

### 2.4 Isolation of EVs

Isolation of EVs was carried out as described.^19^, ^20^ Briefly, cells were grown and differentiated as indicated above in media containing exosome‐depleted FBS. Collection of conditioned media was done following a 24‐h incubation period in DMEM at either normoxic or hypoxic conditions or DMEM containing MCM. Media was centrifuged at 2000 × *g* for 10 min and filtered (0.22 μm) to remove dead cells and cellular debris. The supernatant was centrifuged at 17 000 × *g* for 30 min. The supernatant was centrifuged at 100 000 × *g* at 4 °C for 75 min. The pellet resuspended in sterile PBS and centrifuged again at 100 000 × *g* at 4 °C for 75 min. The final pellet containing small EVs was resuspended in PBS, aliquoted, and stored at –80 °C until use. The same procedure was used for the isolation of plasma EVs, with a minor modification. The plasma was diluted 1:3 volume in PBS prior to the initial centrifugation. EV size distribution and concentrations were determined by nanoparticle tracking analysis (NTA) using a Malvern Nanosight NS300 instrument. EV preparations were aliquoted and stored at –80 °C.

### 2.5 Cryo‐Electron Microscopy

EVs were directly adsorbed onto glow‐discharged holey carbon grids (QUANTIFOIL, Germany) and processed as described.^21^ Images were obtained in a JEM‐2200FS/CR transmission cryo‐electron microscope (JEOL, Japan).

### 2.6 Insulin‐Stimulated 2‐Deoxyglucose Uptake

Cells were washed in Krebs Ringer Hepes buffer (pH 7.4) twice and incubated in this buffer for 45 min. Insulin was added at 100 nm for 15 min (SGBS cells) or 30 min (3T3L1) prior to the assay. 2‐Deoxyglucose uptake media was added containing 2 μCi mL^–1^ of ^3^H‐2‐deoxyglucose and unlabeled substrate at 0.1 mm final concentration. Uptake was allowed to proceed for 10 min (3T3L1 cells) or 15 min (SGBS) and stopped with four washes in ice cold PBS containing 50 mm glucose. Cells were lysed in 0.1 n NaOH and a fraction of lysate counted in scintillation cocktail. An aliquot of lysate was used for protein determination. Measurements were normalized to cellular protein content.

### 2.7 Cellular lysates and Western blotting

Cellular lysates and Western blotting were carried out as previously described.^22^

### 2.8 Extraction of RNA and qPCR analysis

Extraction of RNA and qPCR analysis was carried out as previously described.^22^ Primer sequences used:*Glut1*: Forward (5′‐3′):GCTGTGCTTATGGGCTTCTC; reverse(5′‐3′): CACATACATGGGCACAAAGC; and *Actb*: forward(5′‐3′): CCTGTGCTGCTCACCGAGGC, reverse(5′‐3′): GACCCCGTCTCTCCGGAGTCCATC was used as normalizing gene.

### 2.9 Statistical Analysis

*T*‐test or analysis of variance as indicated in the figure legends, were carried out using GraphPad Prism6 with a confidence interval of 95% and statistical significance was considered if *p* < 0.05.

## 3 Results

### 3.1 EVs from Hypoxic Adipocytes Impair Insulin‐Stimulated Glucose Uptake and AKT Activation in Adipocytes

In obesity, adipose tissue expansion results in local tissue hypoxia and inflammation. These two mechanisms cause cellular stress and thus, could contribute to the generation of EVs by adipose cells. To evaluate the role of the EVs released by adipocytes under these conditions independently, we generated two in vitro models using the well‐established 3T3L1 mouse adipose cell line (Supporting Information Figure S1). For this, cells were either incubated under hypoxia (1% O_2_), normoxia (21% O_2_, control cells), in DMEM media alone, or supplemented with macrophage conditioned media (MCM, 15%v/v) enriched in pro‐inflammatory cytokines. Both conditions caused the adipocytes to become insulin resistant as seen by a decrease in the activation of AKT following insulin stimulation (Supporting Information Figure S1).

**Figure 1:**
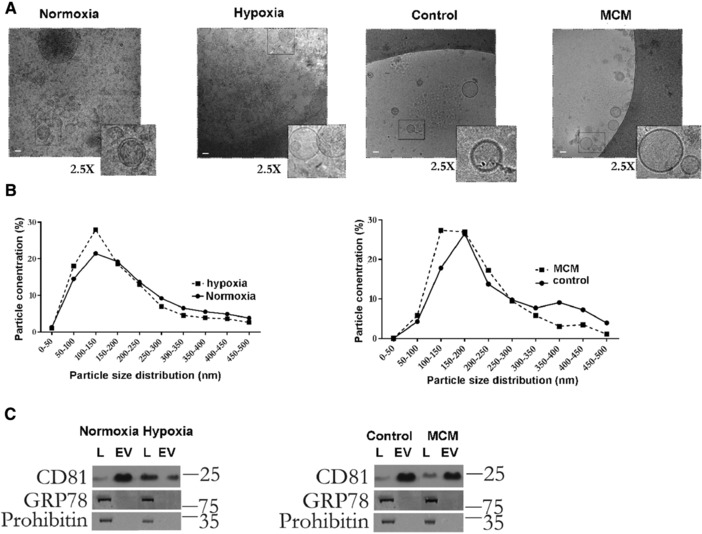
Characterization of EVs released by 3T3‐L1 adipocytes. A) Cryo‐electron microscopy from Control (untreated), hypoxic or cells exposed to macrophage media (MCM). Scale bar: 100 nm. B) NTA of EVs from normoxic/ hypoxic cells and control/MCM‐exposed 3T3L1 adipocytes. C) Immunoblot of cellular lysates (L) or EVs from control, hypoxic, or MCM exposed cells. Equal amounts of lysate or EVs (1 μg) were separated by SDS‐PAGE and immunoblotted with specific antibodies as indicated.

We next isolated EVs released by the cells into the conditioned media and examined EV preparations by cryoelectron microscopy (**Figure** 1A) and immunoblotting (Figure 1C) to confirm purity of the preparations. EV size and concentration was assessed by NTA (Figure 1B). EV size distribution was consistent among experimental groups and consistent with that of exosomes (Figure 1B). EV preparations were enriched in CD81, a marker of exosomes, and markedly devoid of a mitochondrial (prohibitin‐1) and endoplasmic reticulum (GRP78) markers (Figure 1C).

**Figure 2:**
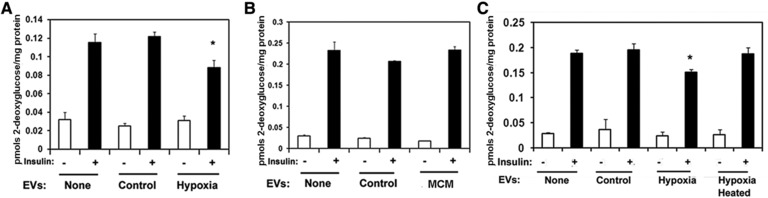
EVs from hypoxic adipocytes inhibit insulin‐stimulated glucose uptake. 2‐Deoxyglucose uptake in 3T3L1 adipocytes that were either left untreated (none) or treated for 24 h with 10 μg of EVs from control or hypoxic adipocytes (A) or with EVs from either control or MCM‐exposed adipocytes (B). The graphs show mean + SEM of a representative experiment and *n* = 3–4 biological replicates. Data is representative of three independent experiments.*indicates *p* < 0.05, One‐way analysis of variance. C) Heat treatment of hypoxic EVs restores insulin stimulated glucose uptake. Adipocytes were left untreated or treated for 24 h with EVs from control or hypoxic cells or EVs from hypoxic cells that were heated 40 °C for 30 min. Following the treatment 2‐deoxyglucose uptake was determined. The graph show mean + SEM, *indicates *p* < 0.05, One‐way analysis of variance (Tukey's test).

To determine the effects of EVs on insulin sensitivity, 3T3‐L1 adipocyte cells were either left untreated or treated with equal amounts of EVs isolated from either normoxic, hypoxic, or cells exposed to MCM. Insulin‐stimulated glucose uptake was measured after 24 h. In untreated control cells insulin increased 2‐deoxyglucose uptake by threefold over basal (**Figure** 2A–C). Cells treated with EVs from control adipocytes exhibited a similar response to untreated cells. However, cells treated with EVs from hypoxic adipocytes displayed a 25% decrease in the insulin‐stimulated response, with no changes in the basal glucose transport (Figure 2A and C). Uptake in cells treated with EVs from cells exposed to MCM was no different than that of untreated cells or treated with EVs from control cells (Figure 2B). Cellular viability was not affected by the EVs treatment (not shown). Heating the hypoxic EV preparations to 40 °C for 30 min prior to the treatment of cells restored insulin‐stimulated glucose uptake (Figure 2C).

**Figure 3:**
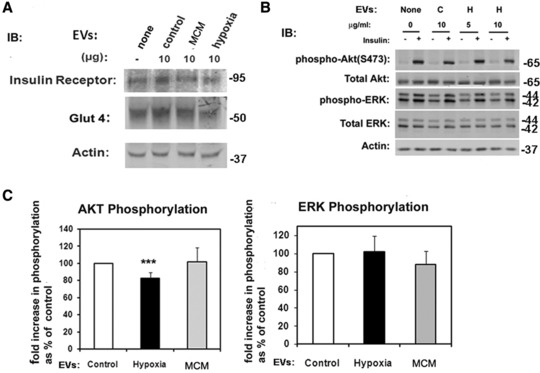
EVs from hypoxic adipocytes inhibit insulin signaling. A) Immunoblot of cellular lysates from adipocytes treated for 24 h with 10 μg mL^–1^ of EVs from control, hypoxic, or MCM‐exposed cells. Equal amounts of lysates were immunoblotted with antibodies as indicated. B) 3T3L1 adipocytes were left untreated or treated with 5–10 μg mL^–1^ EVs for 24 h (C: control, H: Hypoxia), and stimulated with insulin (100 nm, 30 min). Lysates were obtained and immunoblotted with the indicated antibodies. C) Quantification of AKT and ERK phosphorylation. Graphs show mean ± SD EV of *n* = 4 experiments for AKT and *n* = 2 experiments phospho‐ERK, ***indicates *p* < 0.01. One‐way analysis of variance.

To explore the molecular mechanisms, we determined the expression of GLUT4 the main glucose carrier involved in the insulin‐mediated glucose transport, insulin receptor, and activation of the proteins involved in insulin signaling. No differences were observed in the expression of Glut4 or the insulin receptor between cell groups (**Figure** 3A). We determined the activation of the insulin signaling pathway by monitoring phosphorylation of Ser473 in AKT and of Thr201/Tyr204 on extracellular regulated kinases (ERK) p44/p42 in response to insulin following the treatment with EVs. In a dose dependent manner, we detected a small but significant decline in AKT S473 phosphorylation in cells treated with EVs from hypoxic cells compared to untreated or cells treated with EVs from control adipocytes (Figure 3B, C). We did not detect any difference in the activation of the ERK proteins in any of the experimental groups (Figure 3B, C). No differences in the expression levels of AKT or ERK proteins were seen.

### 3.2 EVs from the Plasma of Subjects with Obesity Impair Insulin‐Mediated 2‐Deoxyglucose Uptake

**Figure 4:**
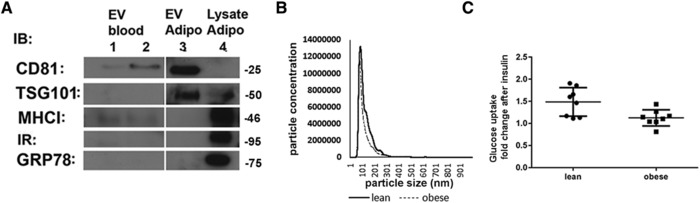
A) EVs from plasma (EV blood), EVs from human adipocytes (EV adipo), and adipocyte cellular lysates were separated by SDS‐PAGE and immunoblotted with antibodies for EV markers as indicated. B) Size distribution of plasma EVs by NTA. C) Insulin‐stimulated 2‐deoxyglucose uptake in SGBS cells treated with 2 × 10^5^ EV mL^–1^ of EVs obtained from lean (*n* = 8) or obese subjects (*n* = 9). Data shows mean ± SD EV, each EV was tested in triplicate in two independent experiments, *p* = 0.0153 (*t*‐test).

To confirm the above data in the context of human obesity, we next isolated EVs from plasma obtained from lean and obese women (Supporting Information Table S1) using previously established ultracentrifugation protocols.^19^ EV samples were analyzed by western blotting to confirm the presence of EV markers (CD81, MHCI) and the absence of other cellular membrane markers (insulin receptor, GRP78) and were compared to EVs released by 3T3‐L1 adipocytes (**Figure** 4A). Plasma EVs contained exosomal markers such as CD81 and MHCI, but were devoid of TSG101 compared to EVs released by 3T3‐L1 adipocytes (Figure 4A). EV particle distribution was similar to the cell models and consistent with that of exosomes, with a peak around 100–150 nm. (Figure 4B)

To preserve any species compatibility that could affect the entrance of EVs into cells, we next examined the effect of these EVs on insulin action in the SGBS human adipocyte cell line. Following differentiation of cells into adipocytes, cells were incubated in DMEM for 24 h to remove hormones and subsequently treated with EVs from either lean women or women with obesity at equal concentration (2 × 10^5^ EV mL^–1^) as determined by NTA, and we assayed 2‐deoxyglucose uptake in response to insulin as described previously.^16^ Transport was normalized to cellular protein content. Insulin‐stimulated glucose transport was decreased in cells treated with EVs obtained from women with obesity (Figure 4C).

## 4 Discussion

Numerous stimuli induce the production of EVs in distinct cellular types. Here, we sought to investigate the hypothesis that EVs released by stressed adipocytes and those circulating in obese subjects could affect insulin action.

The size distribution of our EV preparations was consistent with those found in other systems including the Otsuka Long‐Evans Tokushima Fatty rats^23^ and adipose tissue derived mesenchymal stem cells.^24^

Previous reports found that obesity leads to an increase in EV release in rodent animal models.^15^, ^25^ However, we did not detect any differences in the concentration of circulating EVs found in subjects with obesity (not shown). However, in agreement with^26^ the model of hypoxic 3T3‐L1 adipocytes produced more EVs compared to normoxic cells, a phenomena seen in other hypoxic cell types. Hu et al.^27^ found hypoxia upregulated the expression of FIP4 a regulatory protein of the small GTP binding protein rab11, which is present in adipocytes and has been involved in exosomal release.^28^ Thus, expression of genes regulated by HIF1α and increased rab11‐mediated trafficking could facilitate EV release in adipocytes, however more experiments are needed to confirm this.

EVs from hypoxic adipocytes and also from subjects with obesity impaired insulin‐stimulated glucose uptake in vitro in cultured adipocytes. Our findings agree with those by Kranendonk et al.^29^ who found adipose tissue‐derived EVs caused insulin resistance in hepatocytes inhibiting insulin mediated AKT phosphorylation, with concomitant decrease in the expression of gluconeogenic genes.

Proteomic profiling studies of EVs released by adipocyte cell models have been documented^8^ including that of hypoxic 3T3‐L1 cells^26^ and of animal models of obesity and diabetes^23^ but to date not much information is available for circulating EVs in human obesity. Available literature from in vitro systems suggests that EVs from adipocytes contain protein and RNA species involved in regulating lipid metabolism and adipokine production.^8^, ^9^, ^11^, ^26^, ^30^, ^31^, ^32^

While the precise molecular mechanisms are at present unknown, we provide evidence that EV action implicates at least in part the AKT pathway. The effect of hypoxic EVs on glucose transport was thermolabile, suggesting an enzymatic activity may be responsible for this effect, We found that PTEN, a protein phosphatase that reduces phosphatidylinositol 3,4,5‐triphosphate levels was present in EVs from hypoxic 3T3L1 adipocytes (not shown). Thus, it is plausible that PTEN exported in EVs may be active in recipient cells. Further experiments are necessary to confirm this.

Finally, since adipocytes produce adipokines that modulate insulin action, it is possible that EVs could affect insulin signaling indirectly through an adipokine‐mediated mechanism. This remains to be explored.

To sum up, our study provides evidence that EVs released by hypoxic adipocytes and in human obesity negatively impact on insulin‐stimulated glucose uptake, in part by inhibiting AKT phosphorylation. The precise molecular entities need to be fully elucidated and warrant further investigation.

**Abbreviations**
ERK: Extracellular Regulated Kinase
EVs: extracellular vesicles
MCM: macrophage conditioned media
NTA: nanoparticle tracking analysis
PI3‐Kinase: phosphatidylinositol 3‐kinase
WAT: white adipose tissue

## Conflict of Interest

The authors have declared no conflict of interest.

## Supporting information


**Suppl. Table 1**. Anthropometrical and biochemical characteristics of the study women. BMI: body mass index.

**Supplemental Figure 1**. A) Hypoxia and MCM cause insulin resistance in adipocytes. Immunoblot of cellular lysates obtained from control cells, hypoxia exposed cells or MCM‐exposed cells (24hr). Prior to lysis cells were left untreated or stimulated with insulin. B) Hypoxic cell model. Left: Immunoblot of cellular lysates of 3T3L1 cells exposed to normoxia or hypoxia (1%O_2_) for 24hrs. Right: mRNA quantification of Glut1. Relative quantification ΔΔCt method to actin as reference gene.
