# Supplementary material for: Extracellular Vesicles from Hypoxic Adipocytes and Obese Subjects Reduce Insulin‐Stimulated Glucose Uptake
Source: Mol Nutr Food Res. 2018 Feb 20;62(5):1700917. doi: 10.1002/mnfr.201700917 (PMC5887919; doi:10.1002/mnfr.201700917)
Supplement: Supplementary file 1 — Suppl. Table 1. Anthropometrical and biochemical characteristics of the study women. BMI: body mass index. [file MNFR-62-na-s001.docx]

**Suppl. Table 1.** Anthropometrical and biochemical characteristics of the study women. BMI: body mass index.

| Subject | | Age (y) | | BMI (kg/m2) | | | Fasting glucose (mg/dl) | | | Fasting Insulin (mIU/L) |
| --- | --- | --- | --- | --- | --- | --- | --- | --- | --- | --- |
| 1 | | 32 | | 21.8 | | | 80 | | | 3.8 |
| 2 | | 56 | | 22.4 | | | 110 | | | 4.0 |
| 3 | | 42 | | 24.3 | | | 92 | | | 2.6 |
| 4 | | 35 | | 25.0 | | | 81 | | | 6.5 |
| 5 | | 58 | | 27.4 | | | 94 | | | 4.4 |
| 6 | | 52 | | 26.8 | | | 89 | | | 5.1 |
| 7 | | 28 | | 18.9 | | | 88 | | | 2.1 |
| 8 | | 34 | | 25.8 | | | 71 | | | 5.7 |
| *Mean (Lean)* | | *42* | | *24.03* | | | *88* | | | *4.3* |
| *Std Dev (lean)* | | *12* | | *2.88* | | | *12* | | | *1.5* |
| 9 | 50 | | | 37.6 | | 84 | | | | 4.1 |
| 10 | 35 | | | 38.8 | | 89 | | | | 6.9 |
| 11 | 59 | | | 38.0 | | 77 | | | | 10.1 |
| 12 | 45 | | | 37.2 | | 111 | | | | 10.3 |
| 13 | 46 | | | 36.4 | | 85 | | | | 4.4 |
| 14 | 58 | | | 39.1 | | 90 | | | | 5.1 |
| 15 | 51 | | | 32.9 | 121 | | | | 11.2 | |
| 16 | 37 | | 35.2 | | 81 | | | | 6.2 | |
| 17 | 38 | | 50.3 | | 112 | | | | 9.0 | |
| *Mean (obese)* | *46* | | *38.47* | | *96* | | | | *7.9* | |
| *Std Dev (obese)* | *9* | | *5.20* | | *16* | | | *2.6* | | |
