# Supplementary material for: Extracellular Vesicles from Hypoxic Adipocytes and Obese Subjects Reduce Insulin‐Stimulated Glucose Uptake
Source: Mol Nutr Food Res. 2018 Feb 20;62(5):1700917. doi: 10.1002/mnfr.201700917 (PMC5887919; doi:10.1002/mnfr.201700917)
Supplement: Supplementary file 2 — Supplemental Figure 1. A) Hypoxia and MCM cause insulin resistance in adipocytes. Immunoblot of cellular lysates obtained from control cells, hypoxia exposed cells or MCM‐exposed cells (24hr). Prior to lysis cells were left untreated or stimulated with insulin. B) Hypoxic cell model. Left: Immunoblot of cellular lysates of 3T3L1 cells exposed to normoxia or hypoxia (1%O2) for 24hrs. Right: mRNA quantification of Glut1. Relative quantification ΔΔCt method to actin as reference gene. [file MNFR-62-na-s002.docx]

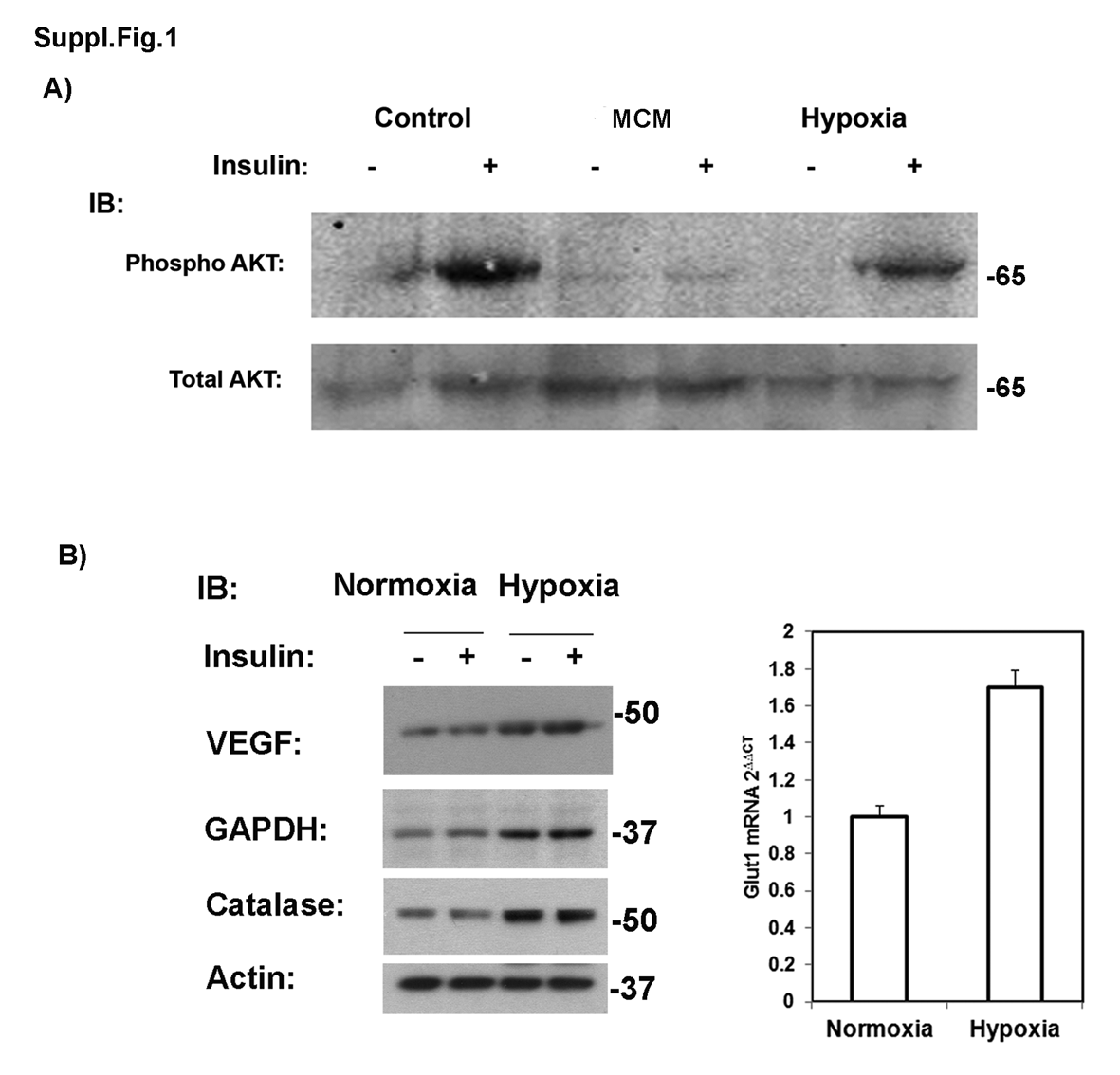


**Supplemental Figure 1.**  A) Hypoxia and MCM cause insulin resistance in adipocytes. Immunoblot of cellular lysates obtained from control cells, hypoxia exposed cells or MCM-exposed cells (24hr). Prior to lysis cells were left untreated or stimulated with insulin. B) Hypoxic cell model. Left: Immunoblot of cellular lysates of 3T3L1 cells exposed to normoxia or hypoxia (1%O_2_) for 24hrs. Right: mRNA quantification of Glut1. Relative quantification ΔΔCt method to actin as reference gene.
